# Empowering Tennessee Pharmacists to Initiate PrEP Using Collaborative Pharmacy Practice Agreements

**DOI:** 10.3390/clinpract13010025

**Published:** 2023-02-15

**Authors:** Alina Cernasev, Rachel E. Barenie, Breanne R. Wofford, Jay Golden, Crystal Walker

**Affiliations:** 1 Department of Clinical Pharmacy and Translational Science, University of Tennessee Health Science Center College of Pharmacy, Nashville, TN 37211, USA; 2 Specialty Pharmacy, Walgreens Company, Nashville, TN 37203, USA; 3 College of Nursing, University of Tennessee Health Science Center College of Nursing, Memphis, TN 38168, USA

**Keywords:** collaborative pharmacy practice agreement (CPPA), PrEP, pharmacist, USA

## Abstract

Background: The uptake of Pre-Exposure Prophylaxis (PrEP) has revolutionized the fight against the Human Immunodeficiency Virus (HIV) epidemic. Consistent obstacles remain that have influenced the slow uptake of PrEP in the United States of America (USA). In order to address these barriers, pharmacists must be included in the dispensing and management of PrEP through collaborative pharmacy practice agreements (CPPAs). Our aim for this study was to characterize pharmacists’ perceptions of initiating PrEP through a CPPA in the state of Tennessee. Methods: This qualitative study was conducted in the USA in 2021 with pharmacists practicing in Tennessee. A framework and specific questions guided the thematic analysis. The words and phrases were coded inductively and later collapsed into categories and placed into emergent themes. Results: Two themes illustrate the voices of practicing pharmacists’ integration in the dispensing and management of PrEP: (1) Learning from other states and previous successful CPPAs to advance and expand innovative models of patient care and (2) advocacy through public policy change to empower pharmacists to initiate PrEP. Conclusion: This qualitative study focused on exploring pharmacists’ perceptions on the opportunity of initiating PrEP through a CPPA in Tennessee. These findings highlight the preparedness of pharmacists to advocate for easier initiative of PrEP in pharmacies across Tennessee, whether through relaxing existing CPPA regulation or pursuing independent prescriptive authority for pharmacists.

## 1. Introduction

The Centers for Disease Control and Prevention (CDC) estimates there are over one million people living with HIV (PLWH) in the United States of America (USA), while the World Health Organization (WHO) approximates there are around 38 million PLWH around the globe [1,2]. The Food and Drug Administration’s approval of Pre-Exposure Prophylaxis (PrEP) medications has served as a positive turning point in battling the HIV epidemic, with oral and long-acting injectable options currently available on the market [3,4]. When adhering correctly to treatment regimens, PrEP is estimated to be around 99% effective in preventing an individual from acquiring HIV via sexual intercourse and is around 74% effective for people who concomitantly inject drugs (PWID) [3,5,6]. Even with this well-documented effectiveness, many patients still experience barriers when they attempt to obtain PrEP, such as stigma within their community, distrust of healthcare providers, lack of financial resources, lack of transportation, and concern for side effects [7,8].

To address barriers to PrEP access and uptake, additional innovative strategies are necessary to overcome existing challenges. Pharmacists should be a key stakeholder considered when developing and implementing new initiatives to increase PrEP uptake due to their accessibility and expertise in medication management. Pharmacists are integrated into various care models, including community settings, ambulatory care, inpatient settings, and more, making them one of the most accessible providers on the healthcare team [9]. In a recent survey study, researchers found that a majority of participants held positive perceptions about pharmacists’ ability to prescribe PrEP, particularly with respondents who reported having a previous experience with a pharmacist, such as through receiving a vaccination or a medication therapy management session [10]. Many states even permit pharmacists to collaborate with a prescriber such as a physician, nurse practitioner, or physician assistant pursuant to an established collaborative pharmacy practice agreement (CPPA). These agreements allow pharmacists to prescribe and dispense certain medications such as PrEP under appropriate and well-defined circumstances [11]. Several states have even taken steps to statutorily broaden pharmacists’ scope of practice and allow them to independently prescribe and dispense PrEP without a collaborative pharmacy practice agreement [12,13,14,15].

Regardless of whether pharmacists are prescribing PrEP independently or under a CPPA, there will likely be a need for additional training for pharmacists that is specific to PrEP to ensure proper implementation [16,17]. Several researchers have previously highlighted that increased training would not only help expand the pharmacists’ knowledge base, but also allow the patient to be more at ease and more willing to trust their pharmacist when providing this service [14,16,17]. Pharmacy-based care, especially in the context of initiating PrEP, is a promising opportunity to improve patient access to evidence-based care and the overall health of the communities pharmacists serve [12,18,19].

Another recent systematic review found a gap in the existing comparative effectiveness data regarding PrEP initiation and continuation through a pharmacist versus a prescriber [20]. The existing studies included in the review found that PrEP access through a pharmacy was a practical and accessible solution supported by most patients; however, long-term effectiveness data regarding this intervention were lacking [20]. Investigators also noted few studies where pharmacists worked under a CPPA in order to provide PrEP services while adding that this was a practical and valued service within the practice of pharmacy [20,21].

Existing data demonstrate that pharmacists’ roles in prescribing PrEP are accepted by patients and would likely address a current gap in patient care. However, pharmacists’ willingness to utilize CPPAs to increase PrEP uptake is not well known. We aimed to characterize pharmacists’ perceptions of initiating PrEP through a CPPA in Tennessee. 

## 2. Methods

### 2.1. Study Design

The research team adopted the theoretical domains framework (TDF) to collect necessary data to achieve the aim of this study [22]. TDF has been used in health research and centers to yield results that facilitate improving healthcare outcomes. This study employed focus groups (FG), which are effective avenues for describing a situation and presenting it through meaningful discussions and context [23,24]. Detailed information regarding the study design was previously published [25]. The Institutional Review Board (IRB) at the University of Tennessee Health Science Center approved this study.

### 2.2. Participants and Recruitment

Participants were recruited based on a list of pharmacists provided by the Tennessee Board of Pharmacy that contained the registered pharmacists’ name, email, and phone number. In order to be eligible to participate in the study, pharmacists were required to be practicing in Tennessee at the time of the study, fluent in English, and available to participate in an online FG. Interested participants contacted the FG’s leaders (AC and CW) who provided a schedule of the possible FG dates. Since data collection occurred during the pandemic, all FG were conducted virtually on a platform with audio and visual capabilities [26,27]. At the beginning of each FG, one of the researchers read the informed consent statement that informed of regarding the purpose of the study, including critical ethical issues including data confidentiality and storage. The participants also had the opportunity to ask questions about the study before they verbally consented. At the end of the FG, the participants received a USD100 gift card. 

### 2.3. Data Analysis 

All the FG were audio recorded and transcribed verbatim by a third party to avoid biases. Transcribed FGs were uploaded into Dedoose^®^ (Manhattan, CA, USA) to facilitate data analysis. To extract the themes from the corpus of transcribed data, Braun and Clarke’s reflexive thematic analysis was employed [28]. After the initial inductive and deductive codes were generated by two independent researchers, the categories were developed and the team met to review the themes [28]. In terms of reflective analysis, the researchers wrote memos during the coding process and used reflective memos throughout the data collection to record thoughts about the FGs and contextual futures that may have occurred during the FGs [28]. 

The aim of this study focused on the FG data that facilitates the extraction of themes centered on the importance of CPPAs and the perspective of implementing it in Tennessee. 

## 3. Results

There was a total of four FGs that included a total of 21 pharmacists practicing in Tennessee. Out of 21, 12 were female, ten were Caucasian, and only one participant identified as Hispanic. Additionally, 14 participants worked in the community pharmacy setting while the remainder was split between hospital, clinic, and home infusion settings. Demographics regarding the participants can be found in Table 1. 

**Figure 1:**
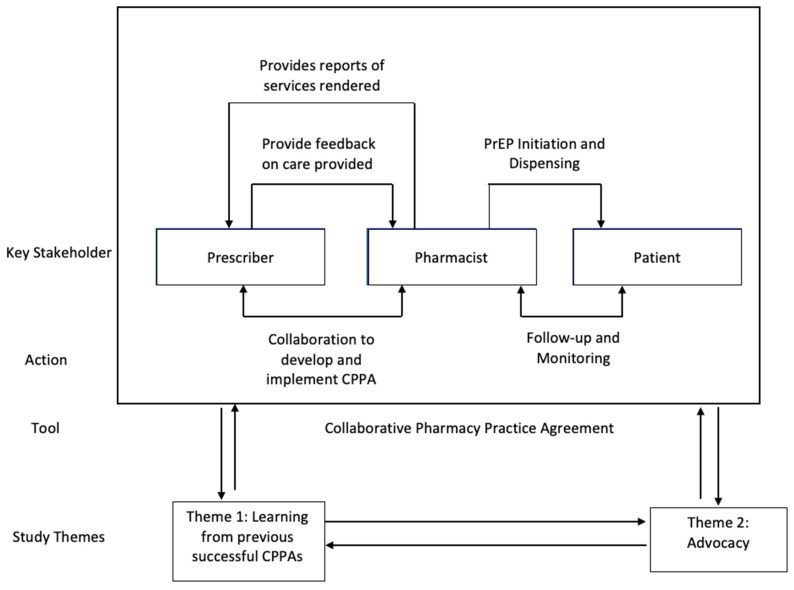
Implementation of a CPPA.

Two themes were identified based on thematic analysis: (1) learning from other states and previous successful CPPAs to advance and expand innovative models of patient care and (2) advocacy through public policy change to empower pharmacists to initiate PrEP. A visual representation of how these themes influence the development and implementation of a CPPA is pictured in Figure 1. 

### 3.1. Learning from other States and Previous Successful Collaborative Pharmacy Practice Agreements to Advance and Expand Innovative Models of Patient Care

Several participants suggested that learning from other leader states such as California and Oregon might be a valuable tool to increase advocacy for possible legislative advancements. For example, one of the participants noted that the legislative model used in Oregon could be used as a standard for other states because of the synergistic relationship created with an interdisciplinary team.

*“…I have read extensively the CPPAs that currently exist. The Oregon model is probably one of the most fascinating to me, having a phlebotomist on staff, and the pharmacy is mainly a PrEP-dispensing clinic. They do other work as well as far as providing pharmaceutical services.”*

(P10, FG10)

The following quotation outlines the benefit that pharmacist-initiated PrEP may have to society since they are the medication experts and have access to various collaborations with other healthcare professionals to enhance patient outcomes. This benefit is presented in the following quote:

*“…I’m just wondering how we would go about that part because PrEP patients have to be tested every three months once they’re started. I think if we were able to do that initial, you know, we have a patient that comes in that’s interested in it, if we can do the initial, let me write you a prescription for this and get them started with the notion that they have to follow up with a clinic to get lab work done, you know, I think that as far as initiating PrEP. I think that that should be shown with the metrics that we have with our metrics to patients, if there was some way that we could show legislation that that’s what we can do, I think it would benefit so many people.”*

(P12, FG3)

The effectiveness of implementing PrEP-specific legislation was compared by one participant to the treatment of an opioid overdose with naloxone. The CPPA that exists in some states for naloxone has essentially streamlined the CPPA process by making it more uniform and requires all pharmacists to work with, for example, the Chief Medical Office of the Tennessee Department of Health, serving as an excellent example of learning from previous CPPAs that have succeeded in expanding patient care. The following extract demonstrates the emphasis of providing data to lawmakers about the success of existing CPPAs to be able to further expand patient care services provided by pharmacists.

*“…So I would guess that a big reason that naloxone has been leaned into so much has been because the financial burden of this has been really obvious to everyone, but I’m not so sure that the financial burden of the HIV epidemic is so obvious in this state, so maybe if that were highlighted to legislators more, then they would be more inclined to promote the PrEP or start initiatives for that.”*

(P21, FG4)

This extract explores the practical value of patient access to PrEP and how to initiate a discussion with them about the advantages of the medication. As patients learn more about HIV, how PrEP works, and the effectiveness of it, they may decide to take it.

*“…just thinking about once we are able to kind of identify that patient population, having strategically placed CPAs, where pharmacists who can have a focus or want to have a focus in improving PrEP intake and uptake can actually take action after that conversation occurs and take that next step with that patient. So that’s kind of where I see the benefit.”*

(P2, FG2)

One participant asserts the value of having access to PrEP from the pharmacy is because of improved accessibility. Being able to pick up PrEP from their local pharmacy would provide flexibility and possibly increased adherence to the treatment for the patient.

*“I think a CPA would just expand access, because, you know, doctors might be super busy, booked up, maybe you can’t get in until-booking three months out or something. Where, versus, the pharmacist running it through CPA, it would just be quicker and more access, easier to get that visit.”*

(P3, FG1)

### 3.2. Advocacy through Public Policy Change to Empower Pharmacists to Initiate PrEP 

This theme presents the participants’ opinions regarding the need to expand pharmacists’ scope of practice to initiate a patient on PrEP without a CPPA or with decreased regulation surrounding existing CPPAs, but one participant noted that the cumbersome and delayed legislative process can be a barrier. He states:

*“…I think for something like that to happen in Tennessee, you would have to have- going back to grass roots-petitions, backing by Tennessee Pharmacist Association [TPA], but also pharmacists, corporations who would be on board to go to board meetings and educate the board and give them examples, here’s what happens in California, this is something that we need to be doing here.”*

(P1, FG1)

This sentiment of overcoming legislative challenges is echoed by P2, FG1, who says:

*“And speaking of barriers, Tennessee, I mean… we all know what the rules are in our state, like it’s extremely burdensome to have a collaborative practice agreement. So I think that that’s a huge barrier.”*

(P2, FG1)

Another participant points out that providing persuasive data is essential when working with lobbyists to move these key legislative efforts forward in the Tennessee General Assembly. She states:

*“…I think we need to focus on lobbying legislators to show them like our statistics, our metrics, how many HIV patients do we serve, show them the Medication Therapy Management [MTMs] that we’re doing and how pharmacist interventions have changed their medications and improved compliance … and to keep them out of the hospital. If we show them with facts and statistics, that will be one of our main things to say, hey, we should be able to prescribe PrEP, and it’s going to be beneficial financially and health wise.”*

(P15, FG3)

There must be a strong partnership between the pharmacy advocacy organizations and the elected representatives of the state to initiate any legislation. This extract clearly states what type of partnership must be created to yield results.

*“…I think our local organizations, and then in concert with the Board of Pharmacy, like I think that we have to have both of those stakeholders onboard to really be advocating for us in politics and in state legislation with the medical board.”*

(P4, FG1)

To have the opportunity to reach a greater number of patients, the discussion highlighted the possibility of expanding current laws to allow for pharmacists to initiate PrEP without a patient-specific diagnosis but still under the authority of a CPPA, similar to opioid antagonists such as naloxone, immunizations, preventative care services, and more. One participant commented:

*”… Just like right now in Tennessee how we have the prescriptive authority for standing orders for like flu vaccines and other such vaccines, maybe giving us some authority as clinical practitioners to be able to prescribe PrEP.”*

(P6, FG2)

This extract accurately captures another obstacle as seen through the lens of a pharmacist who works in a large pharmacy chain.

*“…It’s definitely multifactorial. The first being that, yes, in Tennessee, we have Collaborative Practice Agreements for prevention methods, so we could collaborate with a provider that’s going to take a lot on the individual pharmacist or particular company in order to pursue those. So some of the corporate legal barriers that exist there.”*

(P10, FG2)

## 4. Discussion

Thematic analysis revealed two main themes: (1) learning from other states and previous successful CPPAs to advance and expand innovative models of patient care and (2) advocacy through public policy change to empower pharmacists to initiate PrEP. These findings highlighted the value of learning from existing evidence-based interventions as well as advocating for implementing those interventions in states where they may not currently exist. Our findings highlight that Tennessee pharmacists are ready and willing to implement future potential policy changes in order to better serve their patients, whether through relaxing existing CPPA regulation or pursuing independent prescriptive authority for pharmacists. 

Many states, including Tennessee, already permit pharmacists to prescribe and dispense medications under a CPPA, but the number of pharmacists who practice pursuant to a CPPA, and more specifically prescribe and dispense PrEP, is less clear [11,29]. Prior research has found that states that have passed laws that allow pharmacists to prescribe naloxone are associated with increased dispensing of naloxone in the community pharmacy setting [30]. Additional states have amended their laws in recent years to explicitly authorize pharmacists to prescribe PrEP independently, but whether similar increases in dispensing have followed is not unclear [12]. Evaluation of whether the passage of these laws, notably independent prescriptive authority for PrEP, is associated with increased pharmacist-prescribed and -dispensed PrEP should be evaluated. In addition, our study highlighted how busy prescriber practices are, and that by allowing pharmacists to initiate PrEP, this may create a more efficient means for patients to access evidence-based care versus potentially waiting months for an appointment, which reinforces findings from the previous literature [29]. 

An important barrier identified is the cumbersome and delayed legislative process to advance pharmacy practice. Our findings show there is a disconnect between the Tennessee General Assembly and pharmacists practicing in Tennessee regarding implementation of the CPPA for PrEP. Previous research demonstrated that PrEP initiation is feasible in pharmacies, pharmacists were supportive of offering PrEP, and the majority of patients hold positive views regarding pharmacist initiation of PrEP [10,14]. Our findings align with previous findings and re-emphasize that pharmacists are ready and willing to prescribe and dispense PrEP but are met with existing barriers. Some of the FG discussions highlighted the important role of advocacy organizations, such as state pharmacy organizations, and the critical value that these organizations offer to mediate the conversation between members of the pharmacy profession and the legislature. In a secondary analysis regarding PrEP and scope-of-practice laws, other investigators found that practitioners such as advanced practice registered nurses were more likely to prescribe PrEP in states that defined their prescribing authority under scope-of-practice laws for PrEP [31]. If similar logic is applied to pharmacists, then if more state legislatures could outline pharmacists’ scope of practice to include PrEP initiation, then perhaps increases in prescribing would similarly follow. It would be valuable to follow up this research with a mixed-method study to explore the collective view of Tennessean practicing pharmacists of the possibility of providing PrEP via telepharmacy to receive a more comprehensive perspective.

Figure 1 highlights how the themes identified in this study influence key features of the CPPA process. These themes also synergistically enhance each other, essentially creating a feedback loop where advocacy and change in turn provide more learning and so on. Future research should focus on evaluating existing initiatives and include all key stakeholders, such as prescribers, pharmacists, patients, and policymakers, to enhance effective efforts and eliminate ineffective initiatives. 

## 5. Conclusions

This qualitative study focused on exploring pharmacists’ perceptions about initiating PrEP through a CPPA in Tennessee. Two main themes were identified: (1) learning from other states and previous successful CPPAs to advance and expand innovative models of patient care and (2) advocacy through public policy change to empower pharmacists to initiate PrEP. These findings highlight the preparedness of pharmacists to advocate for easier initiative of PrEP in pharmacies across Tennessee, whether through relaxing existing CPPA regulation or pursuing independent prescriptive authority for pharmacists.

## Figures and Tables

**Table 1:** Participant Demographics.

Gender	Race	Ethnicity	Practice Site	Years in Practice
M	Caucasian	Non-Hispanic	Community Pharmacy	17
M	Caucasian	Non-Hispanic	Hospital	5
F	Caucasian	Non-Hispanic	Clinic	3
F	White	Non-Hispanic	Home Infusion	2
F	Black	Non-Hispanic	Community Pharmacy	4
Not provided	Indian	Non-Hispanic	Community Pharmacy	21
F	Caucasian	Non-Hispanic	Hospital	1
M	Caucasian	Non-Hispanic	Community Pharmacy	10
M	African American	Non-Hispanic	Community Pharmacy	6
M	Caucasian	Non-Hispanic	Community Pharmacy	5
M	African American	Non-Hispanic	Community Pharmacy	14
F	Caucasian	Non-Hispanic	Community Pharmacy	30
M	African American	Non-Hispanic	Community Pharmacy	45
F	Asian	Non-Hispanic	Community Pharmacy	25
F	African American	Non-Hispanic	Community Pharmacy	3
F	Caucasian	Non-Hispanic	Clinic	5
F	Asian	Non-Hispanic	Hospital	3
F	African American	Non-Hispanic	Community Pharmacy	4
M	Caucasian	Non-Hispanic	Hospital	3
F	Asian	Non-Hispanic	Community Pharmacy	1
F	Latino	Hispanic	Community Pharmacy	1

## Data Availability

No data is available due to privacy and ethical restrictions.
